# How to measure premature mortality? A proposal combining “relative” and “absolute” approaches

**DOI:** 10.1186/s12963-021-00267-y

**Published:** 2021-10-26

**Authors:** S. Stefano Mazzuco, M. Marc Suhrcke, L. Lucia Zanotto

**Affiliations:** 1 Department of Statistical Sciences, University of Padova, Via Cesare Battisti 241, 35121 Padua, Italy; 2 Luxembourg Institute of Socio-Economic Research, Maison des Sciences Humaines 11, 4366 Esch-sur-Alzette, Belval, Luxembourg; 3 Centre for Health Economics, University of York, York, UK; 4 Department of Economics, University of Venice, Fondamenta San Giobbe 873, 30100 Venice, Italy

**Keywords:** Premature mortality, Mixture model, Hierarchical model

## Abstract

**Background:**

The concept of “premature mortality” is at the heart of many national and global health measurement and benchmarking efforts. However, despite the intuitive appeal of its underlying concept, it is far from obvious how to best operationalise it. The previous work offers at least two basic approaches: an absolute and a relative one. The former—and far more widely used— approach sets a unique age threshold (e.g. 65 years), below which deaths are defined as premature. The relative approach derives the share of premature deaths from the country-specific age distribution of deaths in the country of interest. The biggest disadvantage of the absolute approach is that of using a unique, arbitrary threshold for different mortality patterns, while the main disadvantage of the relative approach is that its estimate of premature mortality strongly depends on how the senescent deaths distribution is defined in each country.

**Method:**

We propose to overcome some of the downsides of the existing approaches, by combining features of both, using a hierarchical model, in which senescent deaths distribution is held constant for each country as a pivotal quantity and the premature mortality distribution is allowed to vary across countries. In this way, premature mortality estimates become more comparable across countries with similar characteristics.

**Results:**

The proposed hierarchical models provide results, which appear to align with related evidence from  specific countries. In particular, we find a relatively high premature mortality for the United States and Denmark.

**Conclusions:**

While our hybrid approach overcomes some of the problems of previous measures, some issues require further research, in particular the choice of the group of countries that a given country is assigned to and the choice of the benchmarks within the groups. Hence, our proposed method, combined with further study addressing these issues, could provide a valid alternative way to measure and compare premature mortality across countries.

## Background

“Premature mortality” is a highly popular metric of population health, widely used, e.g. for the purpose of international and country-level performance assessments interested in capturing some dimension of an “unnecessary” or “avoidable” burden of mortality. It features prominently, for instance, as a target of Sustainable Development Goal 3 (“Ensure healthy lives and promote wellbeing for all at all ages”).^1^ Quantifying premature mortality can be useful from a policy perspective, too, in that a particularly high level—or a rising trend—in premature mortality may alert policymakers to an underlying population health problem. However, while the concept is intuitively convincing and potentially informative to policy, premature mortality is hard to measure unambiguously, given its latent nature. In practice, several different measures of premature mortality are in circulation. For example, the OECD measures premature mortality in terms of potential years of life lost (PYLL) before the age 70 [2]. Similarly, the Global Burden of Disease Study [3] uses the “Years of Life Lost” (YLL) as its proxy for premature mortality, which is calculated from the number of deaths multiplied by a global standard life expectancy at the age at which death occurs. The WHO considers an age-standardised overall mortality rate from age 30 to under 70 years [4], while Eurostat favours an age-standardized rate below age 65 [5]. In the demographic literature, a very different, more “endogenous” approach is used, distinguishing a distribution of “natural” deaths from that of premature deaths, as first suggested by Lexis [6], and more recently considered by Kannisto [7, 8] and Cheung et al [9]. In this perspective, there is no exogenous age threshold, but only two—partially overlapping—curves of mortality. These two fundamentally different approaches in defining and measuring premature mortality may be referred to as “absolute” and “relative” approaches, respectively. While the “absolute” approach uses a fixed age threshold to distinguish between “premature” and “senescent” deaths, the “relative” approach does not define any age threshold, but derives premature mortality on the basis of the age distribution of deaths.

The advantage (and likely appeal) of the “absolute” approach is in its straightforward implementation and interpretation, once the decision on the age threshold is taken. Yet, it is far from clear how the age threshold should be selected, and—more importantly—the choice of specific threshold may critically determine how countries compare against each other in terms of premature mortality. By contrast, the “relative” approach does not suffer from the challenge of setting an arbitrary age threshold, though at the cost of a more difficult interpretation of what a “mature” or “normal” death, as defined by Lexis [6], really means. Moreover, cross-country comparisons are difficult to implement, since the extent of premature mortality depends on the senescent mortality, which substantially varies across countries.

In this paper, we acknowledge that both approaches have strengths and weaknesses, leading us to propose a third way, which may be considered as a constructive compromise between the two.

### Premature mortality: an absolute view

**Fig. 1:**
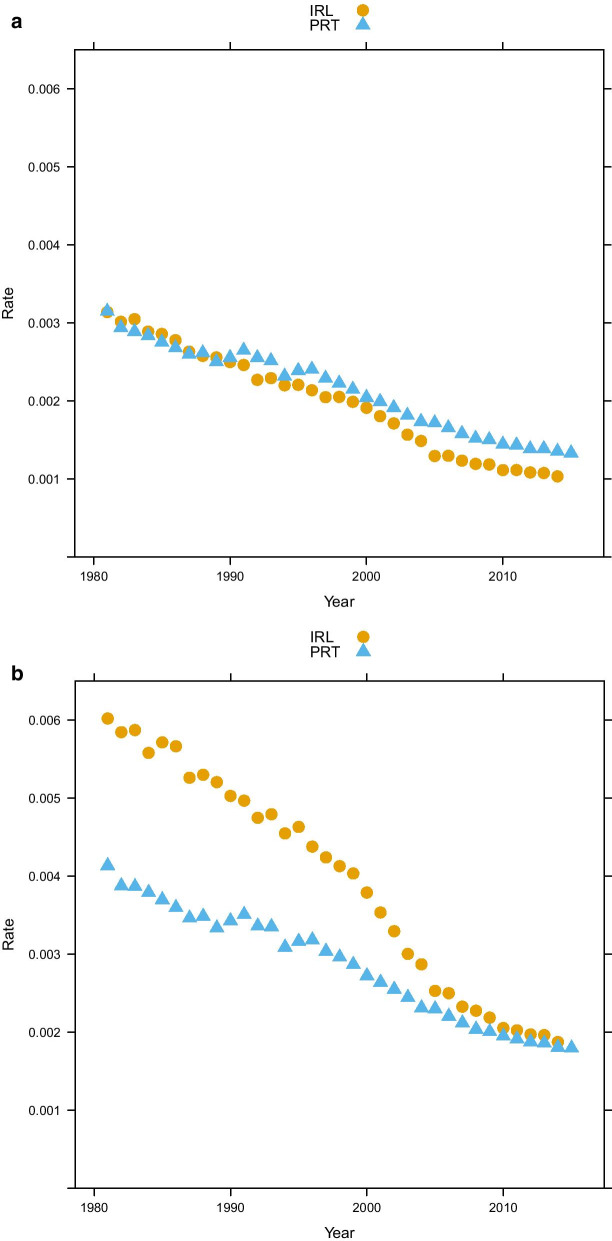
Premature mortality, Ireland and Portugal mortality rates with different age threshold (panel **a:** age threshold: 65, panel **b:** age threshold: 75) (source: Own elaborations from HMD)

The “absolute approach” to measuring premature mortality is the method used by major international institutions that engage in burden of disease and health system performance measurement [e.g. 4, 10, 11]. This approach involves fixing a certain age threshold, below which every death is defined as “premature”. Unfortunately, there is no clear consensus on what this threshold should be: some use 65 years [12], others 70 years and yet others use 75 years of age [13]. Figure 1 illustrates how changing the threshold might well change the ranking of countries: while in Ireland, the below age 75 and below age 65 death rates are very close to each other, and the gap is much greater between the two measures for Portugal. Hence, when using the age 75 threshold, Portugal shows a lower rate of premature deaths than Ireland. This ranking is reversed, if we apply an age 65 threshold. More generally, a fixed cut-off does not take into account the specific features of the overall mortality of a given country: a 65-year threshold might seem inadequate for countries characterized by high life expectancy (e.g. Sweden or Japan), while a 75-year threshold clearly is not suited for countries with a life expectancy close to or below 75 years (e.g. Argentina or Brazil).

Several related to premature mortality concepts are based on fixing an age threshold: “midlife mortality”, for instance, which is sometimes defined as the mortality rate at age 45–54 and has been recently used to show a rising trend in the USA [see 14], while Eurostat uses also “amenable” and “preventable” deaths [see 10, 11]. In essence, amenable deaths capture those causes of deaths that could have been avoided with adequate treatment, while preventable deaths could have been avoided by improved preventive behaviors or measures (i.e. reduced smoking, healthier diets, screening).

A further popular “absolute” measure for premature mortality is the Potential Years of Life Lost (PYLL), calculated by multiplying the number of deaths at each age by the number of potential years remaining for that age. Yet also in this case, an ultimately arbitrary cut-off age has to be selected [15]. A possible choice could be using the average current life expectancy in the given population. However, in a comparative perspective, it would be difficult to choose a different threshold for every country. This has led some authors to use a so-called “standard life expectancy” (SLE) [16], i.e. a life expectancy representing the potential maximum life span at a given age. The value of SLE ranges from 86.01 year (using GBD 2010 study) to (91.93 using WHO Global Health Estimates), see [16]. Although this procedure seems less arbitrary than others, it remains at least debatable whether deaths at ages between 80 and 90 may be considered as truly “premature”, especially in the context of countries with a relatively low average lifespan. This is not merely a philosophical concern: the distinction of premature mortality from senescent one is important from a public health perspective, because the causes and the underlying risks factors associated with them are different. As an example, the rising concern in the US on “deaths of despair” [see 17] derives from analysis of midlife mortality, for which external causes, e.g. suicide, drug and alcohol attributable deaths are particularly relevant. Therefore, while making a distinction between premature and senescent deaths is important, choosing a meaningful age-threshold remains contentious.

### Premature mortality: a relative view

**Fig. 2:**
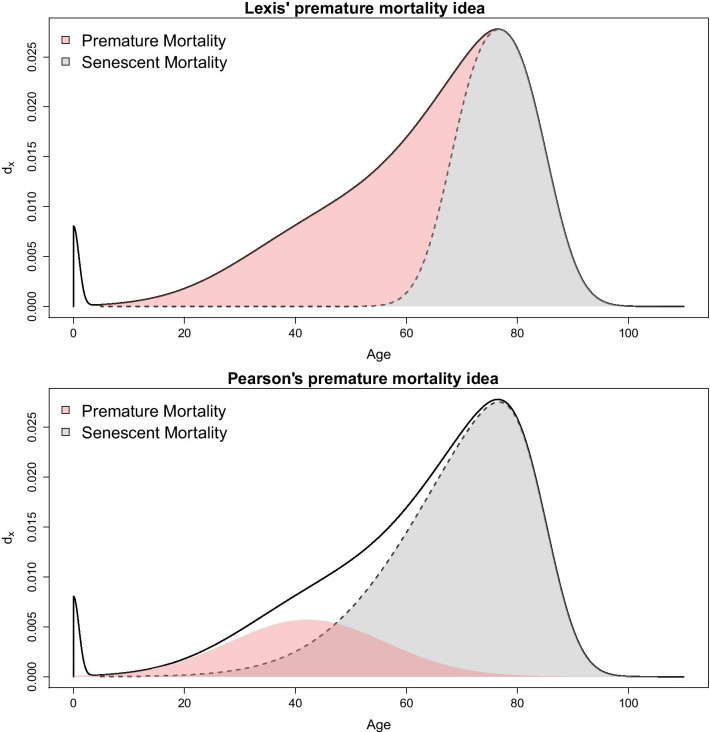
Two views for measuring premature mortality (red area) considering a relative framework

A very different approach to achieve the aim traces back to Lexis [6], who suggested that premature mortality could be measured by considering the age distribution of deaths (i.e. the death counts of the life table): according to Lexis, in the absence of premature mortality, this distribution should have a symmetric shape. Thus, the last, most right-hand part of the curve (from the modal age at death up to the end) can be “unfolded” to the left in order to obtain the hypothetical curve without premature deaths (called “normal deaths” by Lexis). Premature mortality in this sense is then measured as the difference between the actual and the hypothetical curve [see 9]. This may be seen as a “relative” measure, as the share^2^ of “premature” deaths depends on the whole distribution of deaths by age. Lexis’ idea was criticized and further elaborated by Pearson [18], who highlighted that also the hypothetical $d_x$ curve in the absence of premature mortality is not necessarily symmetric. Therefore, he suggested a more complex curve to fit premature mortality. Figure 2 shows graphically the difference between the two approaches.

**Fig. 3:**
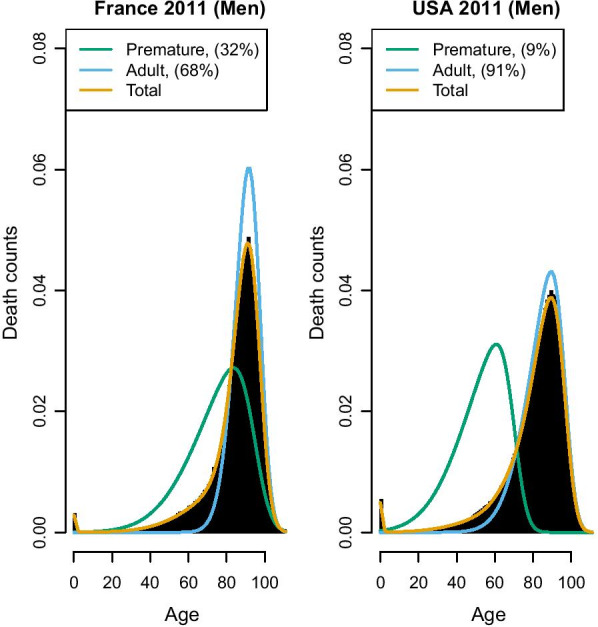
France and USA 2011, Senescent and premature mortality curves using the relative approach (source: Own elaborations from HMD)

**Fig. 4:**
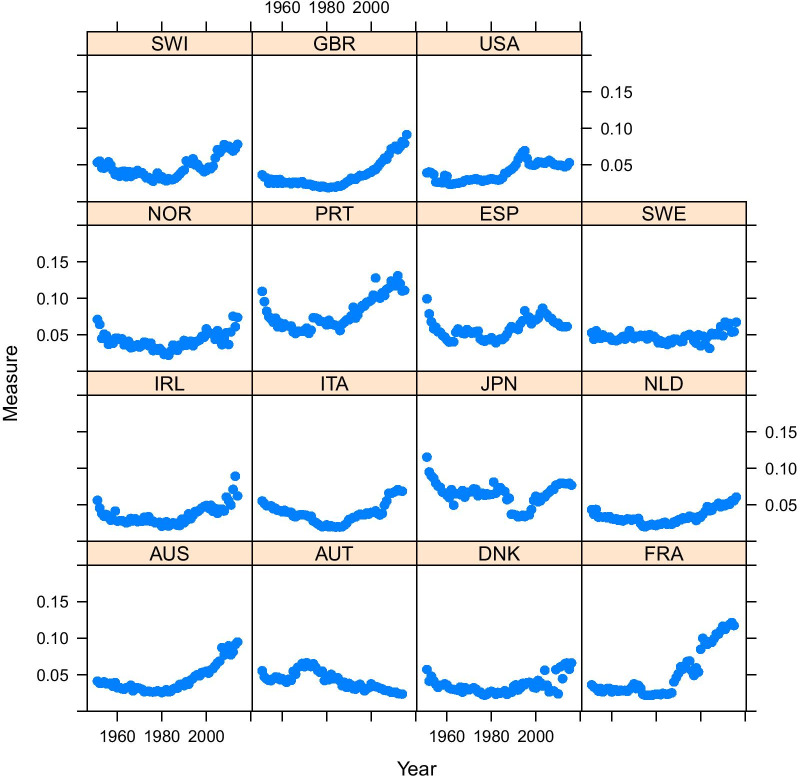
Prevalence of premature mortality in selected high-income countries, using the relative approach (source: Own elaborations from HMD)

**Fig. 5:**
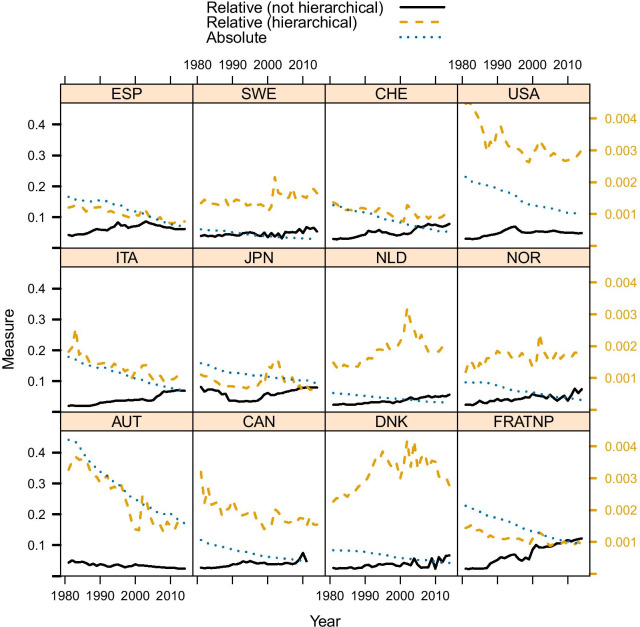
Prevalence of premature mortality in selected countries, using absolute, relative and hierarchical approach (source: Own elaborations from HMD)

More recently, Zanotto et al. [19] implemented a mixture model taking into account Pearson’s reasoning. This model is embedded in an growing literature that considers the life table age-at-death distribution as a convenient function to fit, in the spirit of recent research by Basellini and Camarda [20], Mazzuco et al. [21] and Pascariu et al. [22]. The proposed mixture model comprises three components of mortality:

1 $$\begin{aligned} f_M(x, \xi _M,\omega _M,\lambda _M) &= {} \overbrace{\frac{2}{\omega _M} \phi \left( \frac{x-\xi _M}{\omega _M}\right) \Phi \left( \lambda _M \frac{x-\xi _M}{\omega _M}\right) }^{\text{Senescent mortality}} \end{aligned}$$

2 $$\begin{aligned} f_m(x, \xi _m,\omega _m,\lambda _m) &= {} \overbrace{\frac{2}{\omega _m} \phi \left( \frac{x-\xi _m}{\omega _m}\right) \Phi \left( \lambda _m \frac{x-\xi _m}{\omega _m}\right) }^{\text{Premature mortality}} \end{aligned}$$

3 $$\begin{aligned} f_I(x)& ={} \overbrace{\frac{\sqrt{2}}{\pi } \exp \left( -x^2 \right) }^{\text{Infant mortality}} \end{aligned}$$

Equations (1–3) define two distributions: $f_M$ is the age at death distribution related to old age (henceforth called “senescent” mortality function) and $f_m$, which is the age-at-death distribution of premature mortality. Note that $f_m$ and $f_M$ are defined with two skewed normal distributions, i.e. a generalization of the normal distribution, allowing for skewness [see 23]. These components are then combined to fit the age distribution of deaths, as follows:

4 $$\begin{aligned} d(x) &= {} \eta \cdot f_I(x) \nonumber \\ &\quad+ {} (1-\eta ) \cdot \alpha \cdot f_m(x, \xi _m,\omega _m,\lambda _m) \nonumber \\&\quad+ {} (1-\eta ) \cdot (1-\alpha )\cdot f_M(x, \xi _M,\omega _M,\lambda _M) \end{aligned}$$

The share of premature mortality is given by the estimate of parameter $\alpha$. It should be noted that there exists a significant literature that models mortality by decomposing the mortality age patterns into three components: Siler [24] and Heligman and Pollard [25] already provided such a decomposition with two different models several decades ago. More recently, Basellini and Camarda [26] also proposed a three-component model. However, in none of these cases do the models yield an estimate of premature mortality. The Heligman and Pollard model is more related to accident-attributable mortality, which is a quite different concept, as it is mainly related to external causes of deaths, and it is concentrated in the younger adulthood age range (see Remund et al. [27] for a detailed analysis of this component), while premature mortality is a broader concept, involving more causes of death and older ages. Siler’s model assumes a constant mortality rate during young adulthood, while the Basellini–Camarda model is more flexible, using the Lee-Carter model applied to each component (childhood, early-adulthood and senescent), it nonetheless does not allow for a quantification of premature mortality.

The advantage of the approach underlying model 4 is that defining an age threshold separating “normal” deaths from premature ones is no longer needed, but premature deaths are defined as the number of deaths that let the real distribution of deaths exceed the hypothetical one, as illustrated in Fig. 2.

We coin this the “relative” approach, as the premature mortality share also depends on how old-age deaths are distributed: if they are shifted to the right, deaths on the right-hand side of the mode are more likely to be included in the premature deaths distributions. Thus, in the relative approach, the exact operationalization of premature mortality also depends on the pattern of the “senescent” mortality, and every country has its own pattern of “senescent” and premature deaths distributions. In this way, we avoid defining a universal threshold that might be questionable for some countries. On the other hand, the relative approach implicitly admits that a death can be deemed as premature—and hence “deserving” to be avoided in one country, and not in another one. This may be seen as problematic from an ethical perspective. Yet, there is also a more practical concern to this approach: if we draw a comparison between, for instance, France and USA, we find a result that may seem surprising (see Fig. 3): the share of premature deaths in France is much higher than that of the USA, and increasing in recent years—a result that is at odds with what may be our knowledge about lifespan in these two countries. France is a high longevity country and recent literature shows that premature mortality has declined (rather than  increased) in the last years [28], while it is well-known that the USA has a lower life expectancy and a relatively high level of premature (or mid-life) mortality [14, 29].

The explanation of Fig. 3 is that in France the “normal” deaths shifted to the right in the last decades, and this probably produced the increase in the share of premature deaths (i.e. deaths that before were included among the “normal” ones are now deemed as premature). In the USA, instead, the “normal” deaths distribution is located far more to the left, thus encompassing also deaths that in France would be more likely considered as “premature”. Figure 4 shows that, surprisingly, the relative measure defined by equation (4) suggests an increasing trend of premature mortality (data provided by Human Mortality Database—HMD). Even more striking is that France is one of the countries showing the sharpest increase of premature mortality in recent years, while the USA share of premature deaths has stabilized somewhat.

Hence, while on the one hand, the relative approach eliminates the difficult choice of the age threshold, allowing for better comparison between countries with different levels of life expectancy, this may come at the cost of producing counter-intuitive results. The explanation of the latter is that with this approach, the share of premature deaths depends also on the shape and location of the senescent curve ($f_M$). Hence if $f_M$ has a relatively large variance and low mean (as we observe in the USA), it might be that the “premature” curve is hidden by the senescent one and so underestimated, while in countries where senescent deaths shift to the right and are highly compressed (like in France), the area of premature mortality is isolated and more visible, but probably overestimated.

## Methods

Both “relative” and “absolute” approaches have revealed some pitfalls that render the comparison of premature mortality difficult across countries. In this section, we present an alternative approach that seeks to combine the positive aspects of either previous approach, while trying to avoid their respective drawbacks. To this end, we propose to group some comparable countries and assume that all of them have the same senescent mortality curve, while the premature mortality curve is allowed to vary across countries. This choice is in line with the idea by Li and Lee [30], who apply the Lee-Carter model to a group of populations, allowing each its own age pattern and level of mortality but imposing shared rates of change by age by adding a common factor. This choice may be justified by the rapid diffusion that innovations in the public health sector can have, leading to a relatively swift diffusion of a longevity improvement in one country to others of the same group. Thus, we similarly assume that countries of the same group should have an equal senescent distribution (the common factor), while the premature curve will be country-specific. In this way, the premature mortality of each country will be easier to compare, as they all will be the complement of the same senescent function.

This will be achieved by constructing a hierarchical model, where premature mortality coefficients are allowed to vary across countries, while senescent mortality parameters remain fixed, according  to the equation^3^

5 $$\begin{aligned} d_j(x)= \alpha _j \cdot f^m_j(x, \mu ^m_j,\sigma ^m_j,\gamma ^m_j) + (1-\alpha _j)\cdot f^M(x, \mu ^M,\sigma ^M,\gamma ^M). \end{aligned}$$

Model (5) is estimated with a Bayesian approach, so prior (and hyper-priors) distributions are defined as follows:

6 $$\begin{aligned} \alpha _j&\sim {} \mathcal{U}(0,0.9) \nonumber \\ \mu ^m_j&\sim {} \mathcal{N}(60,\sigma ^2_{\mu ^m})T[-\infty ,75] \nonumber \\ \sigma ^m_j&\sim {} \mathcal{U}(0,20) \nonumber \\ \gamma ^m_j&\sim {} \mathcal{N}(0,\sigma ^2_{\gamma ^m})T[-0.8,0.995] \nonumber \\ \mu ^M&\sim {} \mathcal{N}(87,4) \nonumber \\ \sigma ^M&\sim {} \mathcal{U}(0,9) \nonumber \\ \gamma ^M&\sim {} \mathcal{SN}(-1,0.5,1)T[-0.995,0.995] \nonumber \\ \sigma _{\mu ^m}&\sim {} \mathcal{U}(0,2.5) \nonumber \\ \sigma _{\gamma ^m}&\sim {} \mathcal{U}(0,0.2) \end{aligned}$$

where $d_j(x)$ is the distribution of deaths by age *x* in the life table of country *j*. We use life table *d*(*x*) rather observed deaths because the latter are confounded by the age structure of populations, while the former are standardized with respect to age structure. Here, infant and child mortality is disregarded so $d_j(x)$ is the distribution only of deaths above age 5. $f_m$ and $f_M$ are two skew-normal probability distribution functions with so-called “centered parametrization” [see 31]. These represent the distribution of premature and senescent deaths, which are mixed with mixture parameter $\alpha _j$. It can be noted that while parameters of $f_m$ depends on *j*, parameters of $f_M$ do not, which means that we assume that senescent component ($f_M$) remains fixed across countries, and the premature one ($f_m$) varies across countries.

As far as prior specification is concerned, mean and skewness parameters ($\mu ^m, \mu ^M$ and $\gamma ^m$) are normally distributed, while $\gamma _M$ is skew-normally distributed, standard deviations ($\sigma ^m$ and $\sigma ^M$) and the mixture parameter ($\alpha$, which is our parameter of interest) are uniformly distributed. Note that most of the priors are non-informative, although some of them have been given a low variance to avoid identification and label-switching problems. For the same reasons, we truncated some priors: the mean of premature mortality curve, for instance, cannot exceed 75 and its skewness cannot be below -0.8. It should be kept in mind that the skewness parameter is bounded between -0.995 and 0.995. We did not report results of sensitivity checks we have made, in particular by changing the variance of priors, getting the same results. This was not a surprise, given the high sample size of our data and that it is well known that in these cases the likelihood tends to dominate the prior information. However, the issue of prior sensitivity might be more relevant when the hierarchical model is applied to sub-national units, with lower sample size. In those cases, sensitivity might be higher.

Models have been fitted in R and STAN software, and the code is available at github repository (https://github.com/stefanomazzuco/Premature_Hierarchical).

This approach also overcomes the ethical issue raised in section "Premature mortality: a relative view", since deaths at a given age will be equally considered in different countries, as long as they belong to the same group. This will be further discussed in the next sections.

## Results

**Fig. 6:**
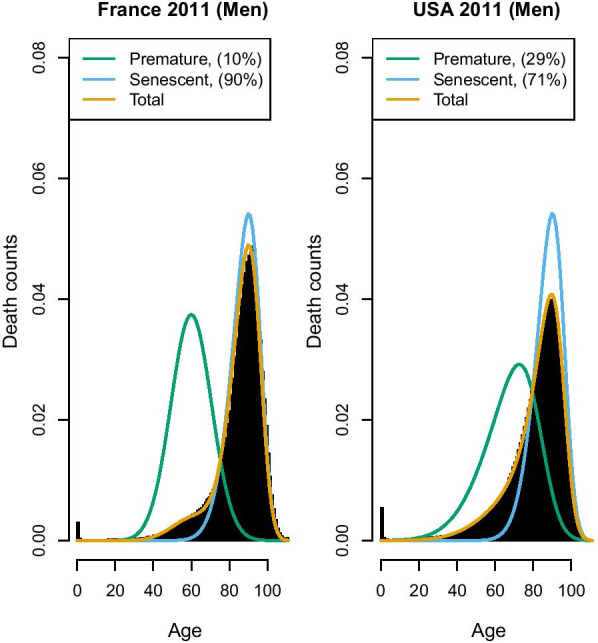
France and USA 2011, Senescent and premature mortality curves using the hierarchical approach (source: Own elaborations from HMD)

We apply this method to data from HMD, considering the countries suggested by Li and Lee [30]. Results are summarized in Fig. 5 and, in addition, the comparison between France and USA is also shown (Fig. 6), which, in contrast to Fig. 3 shows that France has a much lower share of premature mortality compared to the USA. The comparison between Figs. 3 and 6 also shows that two similar fits of observed data can yield two quite different estimates of premature mortality. Hence, the issue of measuring premature mortality is not solely related to goodness of fit, and the definition of the benchmark (i.e. the senescent mortality curve) appears to be far more important. Inspecting the results obtained for all considered countries (see Fig. 5), we notice that using this approach, premature mortality in the USA is much higher than in other countries. Moreover—differently from what was suggested based on absolute and relative measures—it appears that this alternative measure shows a stagnation of premature mortality, which remains at 30% since the beginning of the millennium.

A particularly high premature mortality is recorded also in Denmark and (somewhat lower, yet increasing) in the Netherlands. These results are not too surprising: the high prevalence of premature mortality in the USA is in line with what has been shown by Case and Deaton [14, 29]. We also know that Denmark underwent a stagnation of life expectancy [32] between 1980 and 2000 (in particular for women, but also for men), and a similar one was observed in the Netherlands [33]. Thus, this hierarchical modeling approach not only provides a “reconciliation” between “relative” and “absolute” approaches, but also provides premature mortality results which are arguably more in line with existing research insights on  the specific countries.

### Composition of groups

**Fig. 7:**
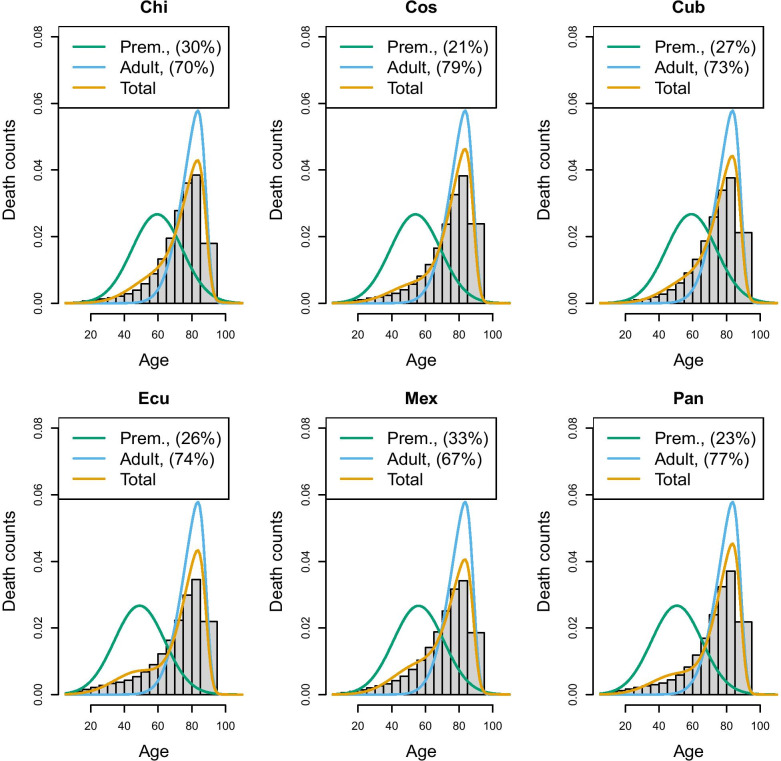
Prevalence of premature mortality in some Latin American countries (high longevity group), hierarchical approach (source: Own elaborations from LAMBdA)

**Fig. 8:**
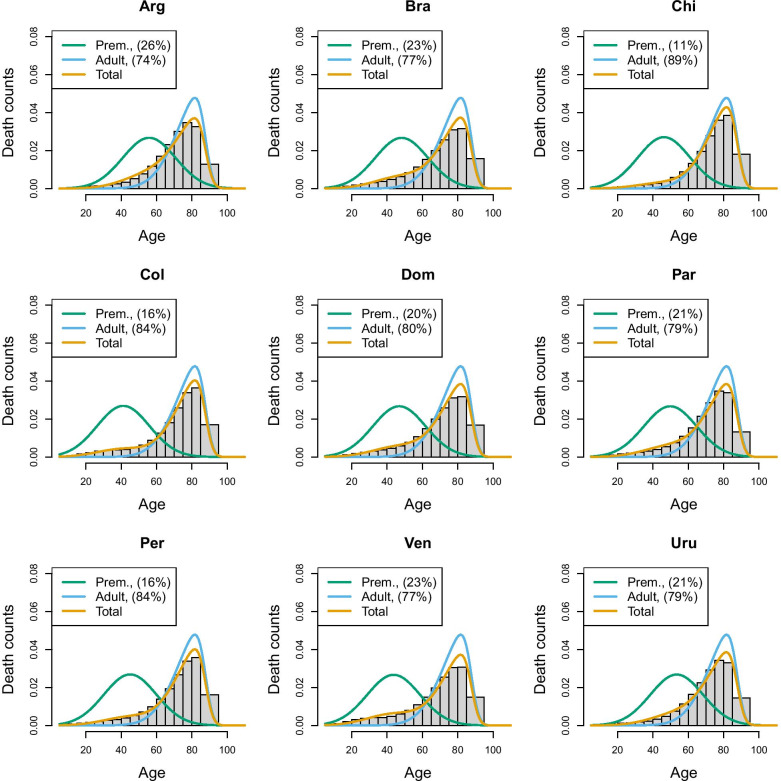
Prevalence of premature mortality in some Latin American countries (medium longevity group), hierarchical approach (source: Own elaborations from LAMBdA)

**Fig. 9:**
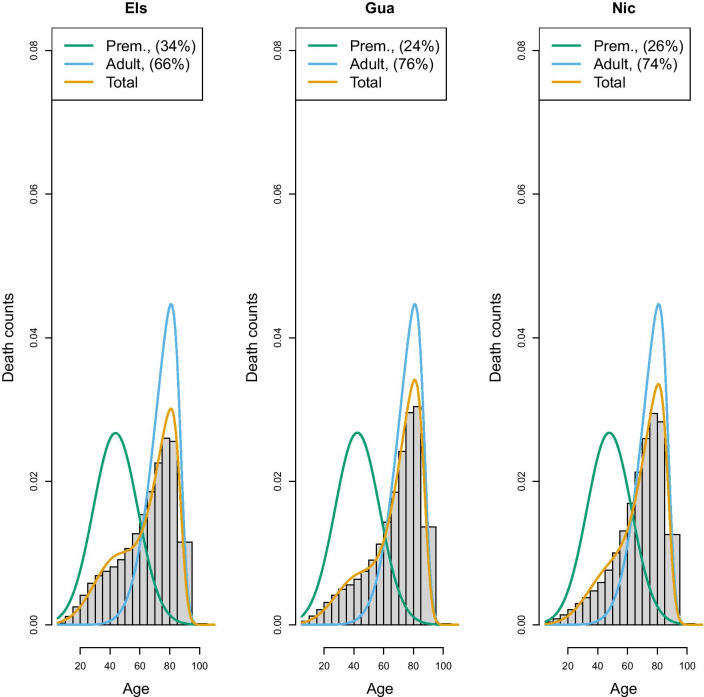
Prevalence of premature mortality in some Latin American countries (low longevity group), hierarchical approach (source: Own elaborations from LAMBdA)

An important choice to be made when implementing the hierarchical model is the composition of the group of countries who will share the same “senescent” mortality. In the above application, we created a group characterized by high longevity, although other choices could also have been taken. To extend the application and to illustrate the challenge of creating groups, we have applied the same model to most recent data of Latin American populations taken from the Latin American Mortality database (LAMBdA) [34]. In this case, groups have been defined by combining countries with (1) a relatively high longevity (Chile, Costarica, Cuba, Ecuador, Mexico, Panama), (2) medium longevity (Argentina, Brazil, Chile,Colombia, Dominican Republic, Peru and Venezuela) and (3) lower longevity (El Salvador, Guatemala and Nicaragua). Figures 7, 8 and 9 show the estimates of the senescent and premature curves. This definition of clusters has also been determined on the basis of the model’s goodness-of-fit to the data: if a country has a mortality profile that is very different from the others in the group, the model would not fit adequately. The reference senescent mortality curves are defined by pooling all the countries of the same group.

In Table 1, we reported also the R-hat statistic [35] for the premature mortality parameter only, and the results confirm a satisfying fit (R-hat statistic with a value around 1 suggest a good mixing of the Markov chain and, therefore, a good fit of the model). However, it is possible to change the composition of the groups keeping an adequate goodness of fit. For example, Chile has been included both in the high and medium longevity group: in the former, Chile has the highest level of premature mortality (29.5%), according to the hierarchical approach, while in the medium longevity group, it has the lowest (10.7%). This means that although statistical measures of fit can assist in a more data driven rather than subjective group assignment, ultimately there will typically remain an element of subjective discretion that can critically affect a country’s relative “performance”. Inevitably, belonging to a high longevity country makes it more challenging to keep premature mortality low, as the senescent mortality is shifted further to the right, while belonging to a low longevity group makes it easier. Therefore, there is no single “perfect” composition of possible clusters, and for any country belonging to a group or another will make the difference in terms of premature mortality estimate. Hence, the choice of grouping will require careful reasoning and transparency. However, this flexibility ensures that countries are not confined to a specific group, but can be compared with different groups. Moreover, after having defined the criteria to create the groups, countries can move from one group to another as their mortality pattern evolves. Hence, this approach does not exclude the possibility that, in the long run, a low longevity country like a sub-Saharian may be comparable to a high longevity country, such as Japan.

### Choosing reference senescent mortality

Another critical choice is about the reference senescent mortality distribution that is used to define the premature mortality distribution. One possibility is to pool all the countries of a given group together and use this “super-country” as the reference—a sort of average distribution. An alternative could be to choose one country as a reference, for example, the country with the highest life expectancy (or the highest modal age at death). Once again, the choice may be partly guided by the goodness of fit: using an average distribution tends to make it easier to have a good fit also for the lowest longevity country, but, on the other hand, having as a benchmark a real country (e.g. Sweden) senescent mortality distribution would facilitate the interpretation of premature mortality prevalence figures, as the “super-population” senescent mortality might be difficult to conceptualise and communicate. There may, however, be cases, in which the “super-population” serves as a very meaningful concept: for example, if sub-national data are considered (regions or provinces), then pooling them together provides a picture of the entire country, and prevalence of premature mortality can be calculated with respect to the national senescent mortality distribution. Hence, the choice of the right reference may be guided by these considerations.

## Discussion

Classifying the previously used approaches into “absolute” versus “relative” ones, we point out limitations in either approach. To overcome those, we propose a hybrid approach that draws out useful elements of both the absolute and relative approach. This new approach assumes senescent mortality to be fixed for all the countries considered as sufficiently “homogeneous”, while the premature mortality curve is allowed to vary across countries. It is hybrid, in the sense that it defines premature mortality *relative* to the benchmark that is chosen, but it is also an *absolute* measure among those countries that are compared with the same benchmark.

**Table 1:** Premature mortality estimates in Latin America countries according to absolute, relative, and hierarchical approach ($\alpha$ estimates)

Countries	$\varvec{\upalpha}$	$\varvec{\upalpha}$	$\mathbf{m_{30-64}}$	Rhat
	(relative)	(hierarchical)	(absolute)	
*Low longevity countries*				
Guatemala	0.372 (3)	0.241 (1)	0.0101 (1)	1
Nicaragua	0.347 (2)	0.263 (2)	0.0104 (2)	1
El Salvador	0.228 (1)	0.335 (3)	0.0127 (3)	1
*Medium longevity countries*				
Chile	0.149 (1)	0.107 (1)	0.0059 (1)	1.02
Colombia	0.251 (6)	0.158 (2)	0.0071 (2)	1.02
Perù	0.215 (4)	0.165 (3)	0.0073 (3)	1.01
Dom. Republic	0.274 (7)	0.203 (4)	0.0082 (7)	1.01
Paraguay	0.223 (5)	0.208 (5)	0.0078 (5)	1.01
Uruguay	0.173 (3)	0.213 (6)	0.0075 (4)	1.02
Venezuela	0.300 (9)	0.226 (7)	0.0089 (9)	1.01
Brazil	0.282 (8)	0.229 (8)	0.0087 (8)	1.01
Argentina	0.170 (2)	0.257 (9)	0.0078 (5)	1.01
*High longevity countries*				
Costarica	0.161 (3)	0.213 (1)	0.0057 (1)	1.02
Panama	0.208 (6)	0.226 (2)	0.0063 (4)	1.03
Ecuador	0.262 (1)	0.259 (3)	0.0073 (5)	1.02
Cuba	0.123 (1)	0.268 (4)	0.0059 (2)	1.05
Chile	0.149 (2)	0.295 (5)	0.0059 (2)	1.02
Mexico	0.237 (5)	0.326 (6)	0.0076 (6)	1.02

Within-group rankings (from highest premature mortality to lowest) are between parentheses (source: Own elaborations from LAMBdA)

As a result, while our hybrid approach overcomes some of the problems of the previous measures, it is not perfect either—and neither could one expect it to be, in light of the latent nature of the concept. Particularly, crucial is determining the criteria driving the choice of the group a given country is assigned to. While statistical testing of model fit can aid in those decisions to a degree, there remains an inevitable degree of arbitrariness that, however, can and should be explicitly addressed and made transparent in its application. For example, Leger and Mazzuco [36] propose a functional clustering of countries’ age distribution of deaths, offering a data-driven way to create groups which are maximally homogeneous, and this—or other equivalent methods, can be used as a starting point for creating country groups. An alternative is to embed a model-based clustering into the hierarchical model, so that premature mortality estimates and groups’ composition are determined jointly. This is certainly an avenue fur future research.

The results we get here look consistent with findings of other scholars who analyzed some specific countries, and there is scope for explaining the trend of premature mortality we get in Fig. 5 for several countries. For instance, the evolution of premature mortality can be explained in terms of evolution of causes of death. This is another avenue of research that will be explored.

## Conclusion

Premature mortality is a latent concept, and as such cannot be truly observed. As we have shown, how the concept is precisely operationalized may well qualitatively affect the results obtained. For instance, a given country may be judged to have done “better” than a different country in terms of one measure of premature mortality, but worse according to another measure. Likewise, in assessing a country’s “progress” in tackling premature mortality over time (e.g. in the context of the SDG progress assessments), that progress may be assessed differently, depending on the exact measure that is employed. As the concept of premature mortality enjoys such (understandable) popularity in international comparisons and benchmarking purposes, it seems critical to use a measure that is as reliable as possible. Our proposed method, combined with further study addressing the clustering issue, could provide a valid alternative way to measure and compare premature mortality across countries.

## Data Availability

The datasets generated and/or analysed during the current study are available in the Human Mortality Database (www.mortality.org) and Latin America Mortality Database (https://www.ssc.wisc.edu/cdha/latinmortality/) repositories.

## Abbreviations

HMD: Human Mortality Database
LAMBdA: Latin America Mortality Database
PYLL: Potential Years of Life Lost

## Footnotes

1 The health target 3.4 is about reducing, by 2030, by one third the premature mortality from non-communicable diseases through prevention and treatment and promote mental health and well-being [1].

2 Note that in this way premature deaths are not individually identified, but only the total share is calculated.

3 For simplicity infant mortality component is disregarded and model has been fitted only on death occurring at age 5 and higher.
