# Correction to: How to measure premature mortality? A proposal combining “relative” and “absolute” approaches

**DOI:** 10.1186/s12963-021-00276-x

**Published:** 2021-11-23

**Authors:** Stefano Mazzuco, Marc Suhrcke, Lucia Zanotto

**Affiliations:** 1 Department of Statistical Sciences, University of Padova, Via Cesare Battisti 241, 35121 Padua, Italy; 2 Luxembourg Institute of Socio-Economic Research, Maison des Sciences Humaines 11, 4366 Esch-sur-Alzette, Belval, Luxembourg; 3 Centre for Health Economics, University of York, York, UK; 4 Department of Economics, University of Venice, Fondamenta San Giobbe 873, 30100 Venice, Italy

## Correction to: Popul Health Metrics (2021) 19:41 https://doi.org/10.1186/s12963-021-00267-y

Following publication of the original article [1], it was reported that the first name of each author was repeated as an initial. The correct authorship list is given in this Correction article and the original article [1] has been updated.

## References

[1] Mazzuco S, Suhrcke M, Zanotto L (2021). How to measure premature mortality? A proposal combining “relative” and “absolute” approaches. Popul Health Metrics.

